# A Behavioral Change–Based Mobile Intervention for Promoting Regular Physical Activity in Medical Rehabilitation Maintenance of Patients With Coronary Artery Disease: Controlled Trial

**DOI:** 10.2196/56480

**Published:** 2024-10-08

**Authors:** Melina Waranski, René Garbsch, Mona Kotewitsch, Marc Teschler, Boris Schmitz, Frank C Mooren

**Affiliations:** 1 Department of Rehabilitation Sciences Faculty of Health University of Witten/Herdecke Witten Germany; 2 DRV Clinic Königsfeld Center for Medical Rehabilitation Ennepetal Germany

**Keywords:** rehabilitation, eHealth, mobile health, mHealth, telemedicine, cardiovascular disease, behavioral change, mobile phone

## Abstract

**Background:**

Cardiac rehabilitation is known to reduce coronary artery disease (CAD) severity and symptoms, but adoption of a healthy postrehabilitation lifestyle remains challenging. Innovative eHealth solutions could help, but behavioral change–based eHealth maintenance programs for patients with CAD are scarce. RehaPlus+ aims to improve postrehabilitation outcomes with a personalized eHealth intervention built on behavioral change concepts emphasizing healthy lifestyle changes, especially regular physical activity (PA).

**Objective:**

This study aims to evaluate the effectiveness of the personalized eHealth program RehaPlus+ for promoting regular PA against usual care.

**Methods:**

A total of 169 patients with CAD who had undergone stent implantation or bypass surgery were recruited after completing center-based phase II rehabilitation. They were then divided, without blinding, into 2 groups using a quasi-experimental approach: a case manager–assisted 24-week eHealth program (RehaPlus+; n=84) and a conventional physician-assisted outpatient program (usual care; n=85). The study was designed as a noninferiority trial. RehaPlus+ participants received motivational messages twice weekly for 6 months, and the usual care group engaged in a 6-month outpatient program (twenty-four 90-minute strength and endurance training sessions). The primary outcomes, evaluated using the self-assessed Bewegungs- und Sportaktivität questionnaire, were regular PA (≥150 min/wk) and weekly activities of daily living (ADLs) 6 months after rehabilitation. Secondary outcomes involved PA during work and floors climbed weekly (measured by Bewegungs- und Sportaktivität questionnaire), psychological well-being (assessed by the 5-item World Health Organization Well-Being Index), cardiac self-efficacy, health-related quality of life (measured by the 36-Item Short Form Survey), and work ability (using the Work Ability Index).

**Results:**

Data of 105 patients (RehaPlus+: n=44, 41.9%; usual care: n=61, 58.1%; male patients: n=80, 76.2%; female patients: n=25, 23.8%; mean age 56.0, SD 7.3 years) were available at the 6-month follow-up. At 6 months after discharge from phase II cardiac rehabilitation, the RehaPlus+ group exhibited 182 (SD 208) minutes per week of PA and the usual care group exhibited 119 (SD 175) minutes per week of PA (*P*=.15), with no interaction effect (*P*=.12). The RehaPlus+ group showed an ADL level of 443 (SD 538) minutes per week compared to the usual care group with 308 (SD 412) minutes per week at the 6-month follow-up, with no interaction effect (*P*=.84). The differences observed in PA and ADL levels between the RehaPlus+ and usual care groups were within the predefined 1-sided noninferiority margin, indicating that the RehaPlus+ intervention is not inferior to usual care based on these outcomes. There were no differences between the groups for all secondary outcomes (*P*>.05).

**Conclusions:**

RehaPlus+ is not inferior to the usual care program, as both groups improved PA and ADLs to a similar extent. These findings emphasize the potential of eHealth interventions to assist in maintaining healthy lifestyles after rehabilitation.

**Trial Registration:**

ClinicalTrials.gov NCT06162793; https://clinicaltrials.gov/study/NCT06162793

## Introduction

### Background

Coronary artery disease (CAD) is a chronic disease without a definitive cure; however, its consequences in terms of restricted physical performance, health-related quality of life (QoL), and perceived well-being can be minimized. Cardiac rehabilitation (CR) is an evidence-based and class IA–recommended therapy [1] provided to minimize the physiological and psychological impact of CAD, decrease morbidity and mortality rates, and enhance physical performance. CR constitutes a complex approach with a strong focus on physical exercise [2] and cardiovascular risk factor reduction, which have been shown to exert the most significant influence on the effectiveness and success of CR [3,4].

**Figure 1:**
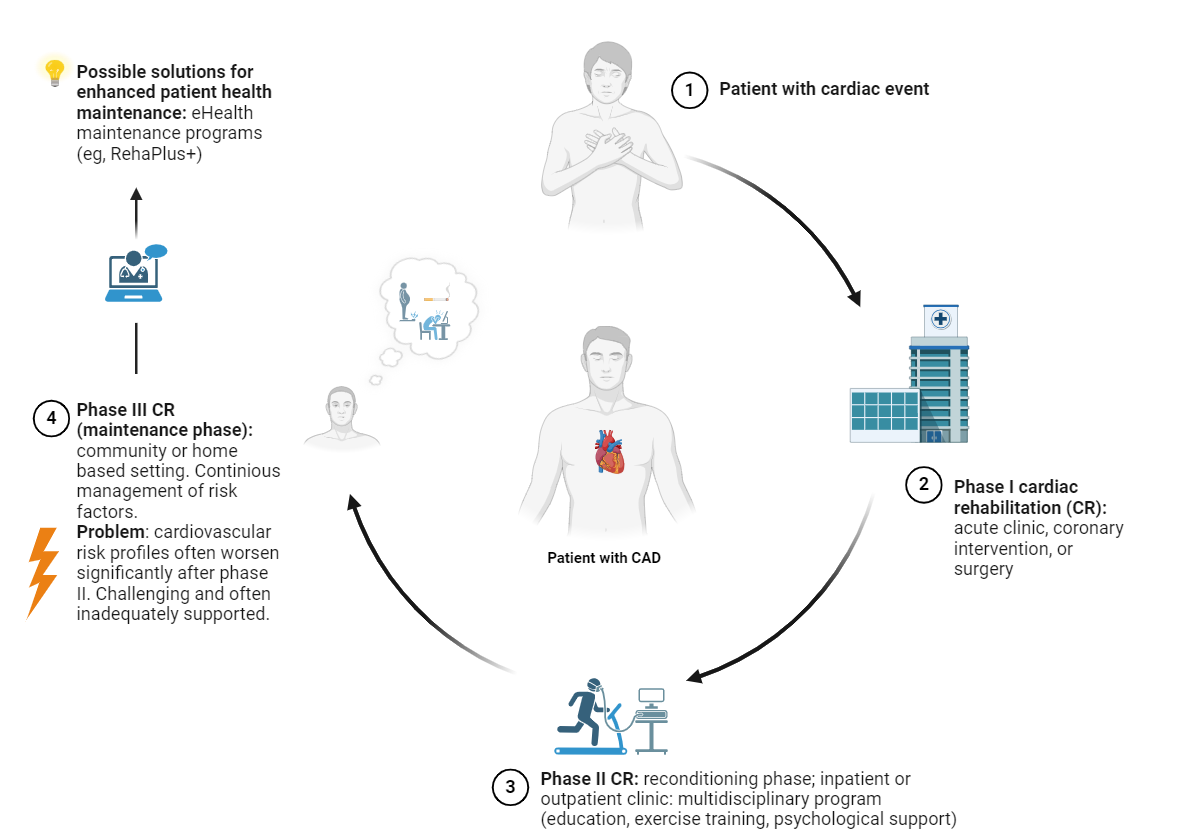
Stages of cardiac rehabilitation (CR). In phase III CR, the usual care program can be prescribed as an outpatient program to patients in Germany. Nearby (<30 km) usual care centers are not always available, leading to many individuals without postrehabilitation program alternatives. eHealth programs such as RehaPlus+ may bridge this gap by providing a readily available alternative for patients with coronary artery disease (CAD).

CR can be categorized into 3 distinct stages (Figure 1) [5]. The initial stage, phase I CR, typically takes place in acute care clinics, often immediately following a coronary intervention or surgery. Patients receive education about their health condition and risk factors, with an emphasis on early mobilization and moderate physical activity (PA) [6]. Phase II CR, the reconditioning phase, takes place in inpatient or outpatient CR centers [7], focusing on patient education, supervised exercise training, diet, smoking cessation, and psychological support [8] to reduce cardiovascular risk, enhance exercise capacity, and support personal health management. Successful completion of phase II CR reduces mortality and morbidity risks [9] and restores the ability to work and engage in social activities [10]. Phase III CR, the maintenance phase, emphasizes lifelong self-care, risk factor management, and regular (self-organized) PA [6]. The successful transition from phase II to phase III CR, including the implementation of healthy lifestyle habits are of paramount significance for the long-term health of patients with CAD [5].

However, and despite the general effectiveness of phase II CR [11], cardiovascular risk profiles often deteriorate significantly thereafter [12]. This effect has been attributed to the fact that maintaining a healthy lifestyle, including regular PA, is challenging for most patients, and adequate support is often not available [13]. Existing maintenance programs are largely affected by high monetary expenses for lifelong support if provided by general practitioners and local cardiologists [14,15]. On the patient’s side, time constraints and travel limitations, impeding participation due to work and family commitments, and general lack of individualization may reduce adherence to center-based care. In addition, during the COVID-19 pandemic, on-site maintenance programs were largely unavailable [16].

Current eHealth solutions for CR maintenance are spurred by innovative technologies and the growing prevalence of mobile devices among patients. The general momentum toward digitalization, including the expansion of mobile data transfer infrastructure, aligns with the concept of mobile health care. This evolution presents significant prospects for enhanced patient health maintenance, as eHealth applications possess the capacity to amplify rehabilitation effectiveness and to sustain patient support postdischarge [17,18]. Prior studies have already provided evidence that electronic communication and health information technology in the form of eHealth may represents an effective alternative to phase II CR [19-21], and a recent meta-analysis on the use of eHealth in phase III CR maintenance suggested that eHealth based on behavior change techniques (BCTs) may assist patients with CAD to achieve improved health outcomes [22]. Most previous eHealth studies in the recent meta-analysis have predominantly used SMS text messages and have not explicitly focused on incorporating BCTs. Hence, this study represents a novelty in this regard, as it is based on behavioral change theories and BCTs, emphasizing the use of individualized messages tailored to the patient’s life situation. A recent study found that a telehealth program may effectively support both behavioral and emotional recovery following a cardiac event [23].

### Objectives

As increasing PA is one of the major goals in phase III CR that has been associated with a 27% to 35% decrease in cardiovascular mortality [24], this study aimed to evaluate the effectiveness of an individualized, message-based, eHealth maintenance concept (RehaPlus+) for the motivation of patients with CAD toward increased PA. Our hypothesis was that RehaPlus+ would be equally effective as the German usual care in form of a center-based maintenance program (Individualized Rehabilitation Aftercare in Post-Acute Treatment; IRENA) in terms of supporting regular PA and activities of daily living (ADLs) as well as \ improvement in psychological well-being, cardiac self-efficacy (CSE), health-related QoL, and work ability assessed 6 months after discharge from phase II CR.

## Methods

### Study Design and Participants

#### Overview

To compare the effects of the eHealth maintenance program RehaPlus+ to the German usual care in the form of a center-based maintenance program (IRENA, provided by the German pension fund), a quasi-experimental study with a comparison at 6-month follow-up (post phase II CR) was performed (Clinical Trials NCT06162793) in accordance with the eHealth CONSORT (Consolidated Standards of Reporting Trials) guidelines (Multimedia Appendix 1). RehaPlus+ has been developed by the Clinic Königsfeld to provide a multimodal eHealth-based aftercare program to patients with CAD as an alternative to the standard rehabilitation aftercare concept. Main outcomes assessed were PA and ADLs. Data for both groups were collected at 2 time points: at the beginning of inpatient CR (baseline; defined as T_0_) and at follow-up (24 weeks after discharge; defined as T_1_). Both groups transitioned to phase III CR within 1 or 2 weeks after discharge from inpatient CR.

#### Eligibility Criteria

**Figure 2:**
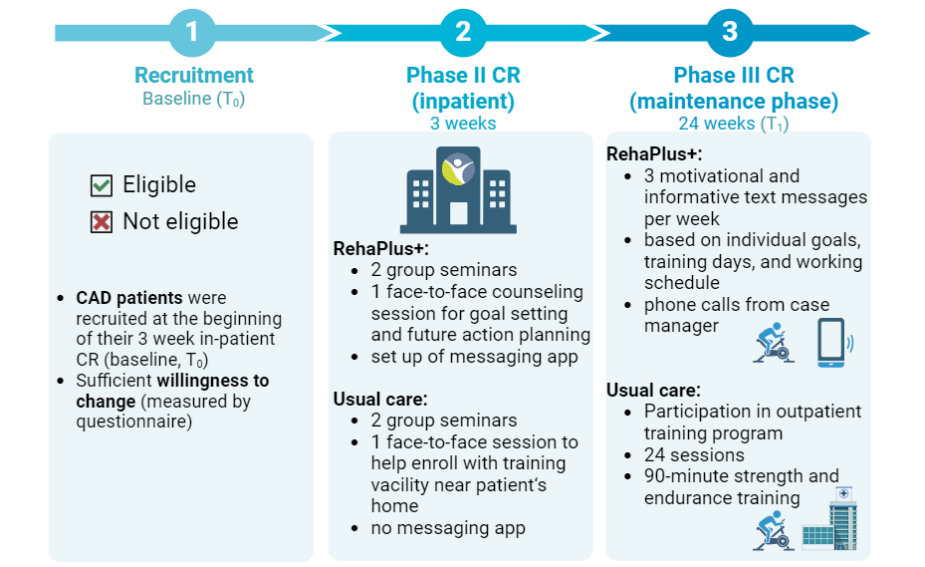
Study design. Patients were recruited at the beginning of the 3-week inpatient cardiac rehabilitation (baseline, T0) and participated in the RehaPlus+ or usual care program, depending on local availability after discharge (24-week maintenance phase). Only patients indicating willingness to change were included. The RehaPlus+ and usual care group attended 2 group seminars and 1 face-to-face individual counseling session in phase II cardiac rehabilitation (CR). In phase III CR, RehaPlus+ recipients received 3 customized motivational text messages weekly for 6 months, while the usual care group engaged in a 6-month outpatient program involving 24 sessions of 90-minute strength and endurance training. Follow-up examinations were conducted 24 weeks after discharge (T1). CAD: coronary artery disease.

The study enrolled patients with documented CAD during inpatient CR (Figure 2). The patients were recruited on-site at Clinic Königsfeld during phase II CR. Enrollment window was within 6 weeks of a cardiac event or intervention, including ST-elevation myocardial infarction and non–ST-elevation myocardial infraction, and stent implantation, and bypass surgery and their combinations. Patients had to declare readiness for behavioral change during phase II CR (see Group Allocation section for details). The patients were required to have the necessary computer and internet skills (assessed in a one-on-one interview) and possess a smartphone. Patients with significant language barriers were not eligible to participate.

#### Ethical Considerations

The study complied with the Helsinki Declaration on Ethical Principles for Medical Research Involving Human Subjects, received approval by the ethics committee of University Witten/Herdecke (#91/2018), and was performed at the medical rehabilitation center Clinic Königsfeld in Germany. Before enrollment, all participants provided written informed consent. The patients had the option to withdraw from participation at any time. The data were deidentified, with patients being assigned numerical identifiers. No compensation was provided to the patients for participation.

### Group Allocation

A quasi-experimental design, as described in the study by Axelrod and Hayward [25], was chosen due to practical and ethical considerations, given that IRENA is regarded as an effective aftercare concept. This approach enables the examination within the constraints of a real-world setting, where full random allocation is not viable.

Both groups (RehaPlus+ and usual care) participated in 2 group seminars during phase II CR (Figure 2). The patients were invited to the initial group seminars based on the transtheoretical model (TTM) of behavior change [26-28], where intentional health-relevant behavior change is categorized into different (subsequent) stages (precontemplation, contemplation, preparation, action, maintenance, and termination). As action-oriented programs are unsuitable for individuals in stage 1 or 2 [28], only patients in at least stage 3 were included. As RehaPlus+ was an experimental program that could not be actively requested by patients, patients with CAD eligible to participate were screened between September 2021 and May 2023 using a questionnaire based on the TTM to determine their readiness for behavioral change [29]. Readiness for behavioral change was congruently accepted by participation in the usual care program, as patients needed to actively request participation by making an appointment with an assistant and connecting with the providing center.

For the individual counseling sessions, both patient groups registered voluntarily. Patients were then assigned to either usual care or the RehaPlus+ program based on the availability of the program in their residential area (30 km radius). Patients with no access to a local center or those who were unable to participate in the program due to occupational reasons were assigned to the RehaPlus+ program. The key difference between the groups was that RehaPlus+ participants received additional support through an app and phone calls in phase III CR, which included personalized messages and action planning, while usual care took part in the usual care program.

### Intervention (RehaPlus+)

#### Overview

The group seminars were rooted in various psychological theories of health behavior change [27,30,31] and concepts of self-efficacy for promoting healthy habits [32]. The initial 60-minute seminar (≤15 participants) focused on a health psychological intervention and aimed at elevating participant’s self-efficacy expectation through action planning, including setting of goals and creating strategies to achieve them. The importance of health-promoting lifestyle changes, including regular PA and ADLs, was explained. Participants outlined their health goals and motivations for altering their lifestyle into a structured action plan. During a subsequent 30-minute seminar, coping strategies tailored to distinct circumstances of each participant were discussed. The health psychology group seminars aimed to empower patients to take independent action and enable them to autonomously pursue and achieve their health-related goals. During the individual face-to-face counseling session, participants received their individual access to the application and were guided through the process of installing the messenger app on their smartphone, and a comprehensive overview of the associated procedures was provided. For individualization of prompts and messages, habits, identified problems or challenges, work commitments, shift schedules, and aspirations were documented and aligned with the patient’s action plan, emphasizing on its feasibility and practicality.

At the end of phase II CR, the eHealth intervention started for a period of 6 months. The RehaPlus+ participants began receiving motivational and informational messages (3 per week using a mobile phone app; described in the App and Messaging section) tailored to their individual objectives, training schedule, and working commitments. Participants were contacted by their designated case manager at 2 and 5 months via telephone to address any technical challenges and barriers encountered in incorporating physical exercise into their daily routine.

#### App and Messaging

**Figure 3:**
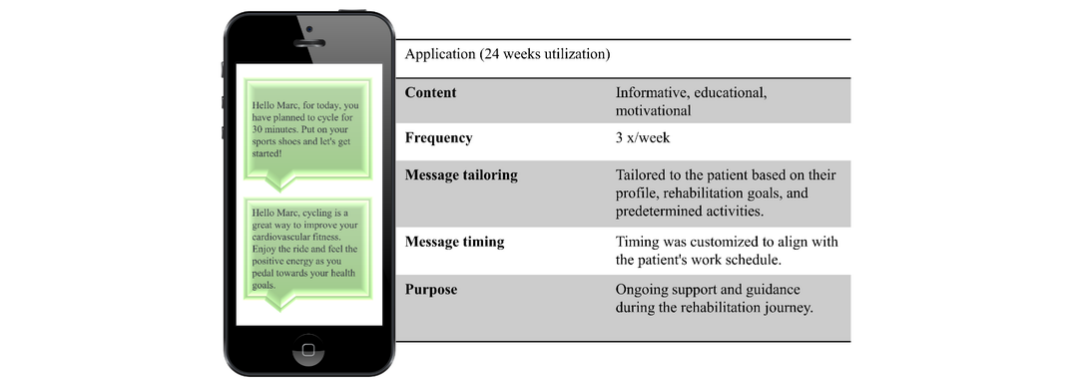
Sample message. Each RehaPlus+ patient received a total of 72 messages over a period of 24 weeks.

An app was developed for the unidirectional delivery of informative, educational, and motivational messages (Figure 3). The messages were sent using end-to-end encryption technology, and patients used a pseudonym to register. No personal data were exchanged via the app. Messages were delivered 3 times per week, with a total of 72 messages within 24 weeks.

The creation process of the messages followed a Delphi approach, involving iterative feedback loops with patients (n=56). Initially, the case manager developed message templates based on common activities. Subsequently, through rounds of anonymous feedback from a panel of patients (n=5), adjustments were made to ensure the messages resonated with their preferences and needs.

Building upon insights gained through the Delphi method, it was identified where individualization would be most beneficial. Messages were then customized accordingly, tailoring them to each patient’s specific needs, defined activities, scheduled timing, and considerations such as working hours. Each message was crafted to focus on specific rehabilitation objectives and suitable PAs. This personalized approach ensured that messages were delivered at optimal times to align with the patient’s schedule, providing continuous encouragement and guidance throughout the rehabilitation process. Messages were intended to provide continuous encouragement and guidance throughout, delivering nudges and support. As an example, a message reads as follows: “Hello [pseudonym], for today, you have planned to pedal for 30 minutes. Put on your sports shoes and let’s get started! Remember, cycling is a great way to improve your cardiovascular fitness and strengthen your leg muscles. Keep up the fantastic work!” The back-end of the system offered a multimessaging functionality, allowing case managers to prepare predefined messaging queues, which were then triggered automatically.

### Usual Care (IRENA)

In Germany, the phase II CR center assists the individual patient in finding suitable postrehabilitation facilities offering the usual care program. It is essential that these recommended postrehabilitation services are located within a convenient distance from the patient’s residence (<30 km) [33]. The usual care program IRENA is a multimodal postrehabilitation program that comprises 24 sessions conducted in small groups with a maximum of 10 participants and begins the end of phase II CR for a period of 6 months. Each session includes a 90-minute combination of strength and endurance training as well as information, motivation, and education, as described in the specifications of the German pension fund [34]. The program includes both an initial assessment and a concluding discussion.

### Blinding

Due to the nature of the intervention, blinding was not possible.

### Outcomes

#### Primary Study Outcomes

Primary study outcomes were defined as self-reported PA and ADLs at 24 weeks (6 months) after discharge, assessed by questionnaire at 4 weeks before rehabilitation (baseline) and 6-month follow-up (see the Questionnaires section). Secondary study outcomes included PA at work and floors climbed per week at 6-month follow-up as well as change in psychological well-being, CSE, health-related QoL, and work ability from 4 weeks before rehabilitation to 6-month follow-up, all assessed by a questionnaire.

#### Questionnaires

PA and ADLs (including PA at work and floors climbed per week) were assessed by the validated German Bewegungs- und Sportaktivität questionnaire [35]. Health-related QoL was assessed through the validated German version of the 36-item Short Form Survey (SF-36; 4-week version; Cronbach α=0.87-0.89) following the guidelines of the RAND Corporation. The SF-36 physical and mental component subscores (physical component summary [PCS] and mental component summary [MCS]) were calculated according to the study by Ware et al [36]. Subjective psychological well-being was assessed using the validated 5-item World Health Organization Well-Being Index (Cronbach α=0.92) [37]. Work ability was evaluated using the validated [38] Work Ability Index (Cronbach α=0.58-0.77) [39]. CSE was measured using the validated CSE Scale (Cronbach α=0.87-0.90) [40]. All outcomes were self-assessed through web-based questionnaires.

### Statistical Analyses

Statistical analyses were performed using SPSS (version 28; IBM) and GraphPad Prism (version 10; GraphPad Software). Constant variables are expressed as mean (SD), median (range), or 95% CI as indicated. Categorical variables are presented as n (%). The normal distribution was statistically and graphically tested via Kolmogorov‐Smirnov test. Differences between groups over time (RehaPlus+ vs usual care) were analyzed using 2-way ANOVA. Multivariate ANOVA with 2 measurement time points, with gender and group as between-subject factors, was conducted to assess the difference between women and men. Categorical variables (gender, education, occupation, and marital status) were analyzed using chi-square test. Data were analyzed for outliers using the robust regression followed by outlier identification option under GraphPad Prism. Data points falling outside the 1% threshold were considered outliers and potentially excluded from the analysis. Cohen *d* was used to express effect sizes. Power calculation was performed using G*Power (version 3.1.9; G-Power). Sample size was calculated based on unpublished data on PA in patients with CAD assessed by Bewegungs- und Sportaktivität questionnaire with an effect size of *d*=0.4 and a noninferiority limit of half the SD, suggesting that a sample size of 100 participants (50 per group) would result in a statistical power of 1–β=0.80 at α=.05. A noninferiority comparison between the RehaPlus+ group and the usual care group was conducted using a predefined margin, assessed by calculating the 95% CI around the primary end point. The noninferiority margin was set at half the SD of 87.5 of the primary end point to represent a clinically acceptable difference between the new intervention and the established standard therapy. According to this approach, a difference less than this noninferiority limit between the groups is considered irrelevant (not meaningful) [41]. The 1-sided interpretation of results was based on the meaningful differences. Responder analysis was performed for PA, ADLs, PA at work, and floors climbed per week, as described using the typical error (TE) method and the following equation: TE=SD_diff_/√2, where SD_diff_ is calculated as the difference between the variance (SD) of 2 repeated measures [42]. Responders were defined as participants who demonstrated an increase greater than 2×TE away from 0. Statistical significance was accepted at *P*<.05.

## Results

### General

**Figure 4:**
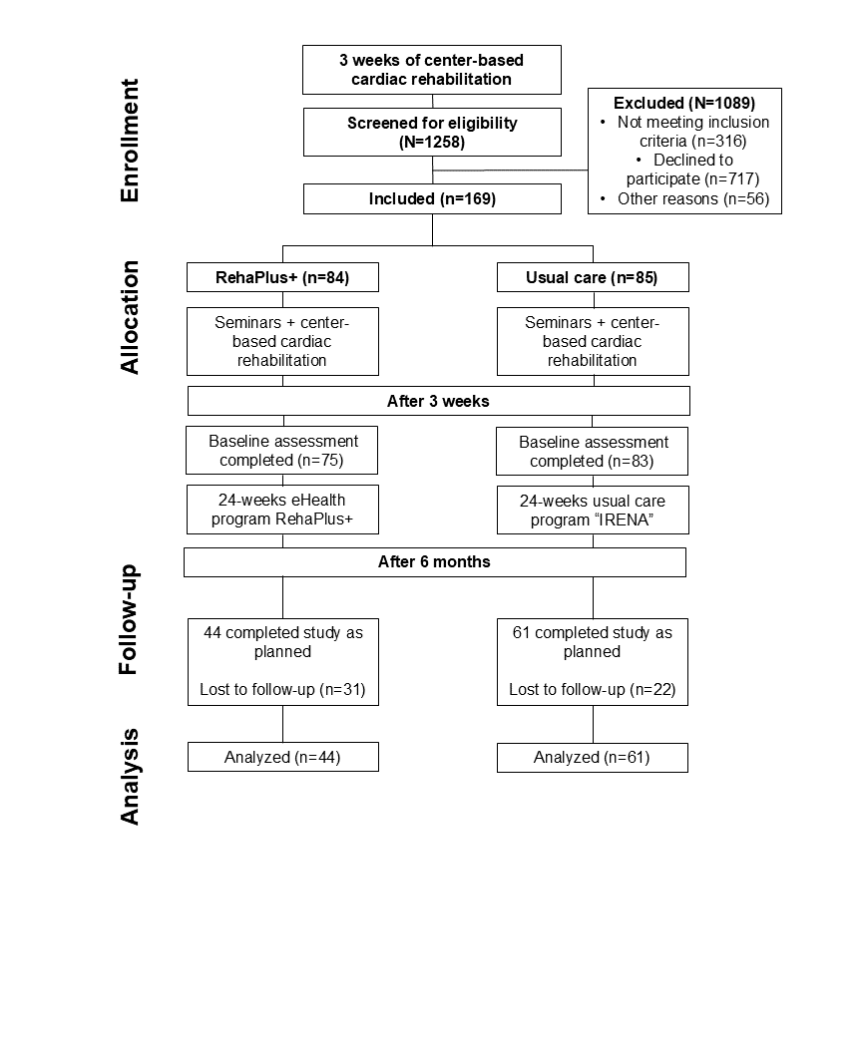
CONSORT (Consolidated Standards of Reporting Trials) flowchart. A total of 169 patients referred to either a 24-week eHealth group (RehaPlus+, n=84) or a conventional center-based program (usual care, n=85). A total of 11 patients (RehaPlus+, n=9; usual care, n=2) did not complete the baseline assessment due to premature discharge. A total of 31 patients and 22 patients were lost to follow-up due to contacting problems for RehaPlus+ and for usual care, respectively. The final analysis included 105 patients (RehaPlus+, n=44; usual care, n=61). IRENA: Individualized Rehabilitation Aftercare in Post-Acute Treatment.

A total of 1258 eligible patients with CAD were screened and 169 (13%) were included (refer to the flowchart in Figure 4). Participants’ baseline characteristics are presented in Table 1. Following a 3-week period of inpatient CR, patients transitioned to the long-term follow-up phase. After 6 months, 41% (31/75) of the RehaPlus+ group and 27% (22/83) of the usual care group were lost to follow-up (*P*=.07) mainly due to contacting problems for follow-up examinations. The final analysis included 105 patients with assessments at both time points (RehaPlus+, n=44; usual care, n=61). During the study, a total of 72 individual messages was sent to each patient in the RehaPlus+ group as planned (n=72, 100%). In general, patients indicated that the messages were motivating and that they served as helpful reminders for performing regular PA. In addition, the handling of the messaging system was found to be straightforward and user-friendly by the case managers, further contributing to its efficiency in supporting patient engagement.

**Table 1:** Patient characteristics^a^.

Characteristics						Overall (n=105)			RehaPlus+ (n=44)			Usual care (n=61)			*P* value
**Anthropometric data**															
	Age (y), mean (SD)				54.9 (7.2)			53.6 (8.1)			56.2 (6.2)			.06	
	**Sex, n (%)**														
		Female		25 (24)			12 (27)			13 (21)			.47		
		Male		80 (76)			32 (73)			48 (79)			—^b^		
	Height (cm), mean (SD)				177.5 (9.1)			179.3 (9.1)			176.0 (8.9)			.06	
	Weight (kg), mean (SD)				94.1 (18.2)			98.2 (16.6)			90.7 (18.9)			*.03* ^c^	
	BMI (kg/m^2^), mean (SD)				29.8 (5.3)			30.6 (5.2)			29.2 (5.4)			.14	
	**Education^d^, n (%)**														
		Less than high school		73 (94)			22 (67)			51 (94)			*.002*		
		Greater than or equal to high school		13 (6)			10 (33)			3 (6)			—		
	**Occupation^e^, n(%)**														
		Worker		22 (22)			8 (19)			14 (25)			.53		
		Employee		71 (71)			33 (77)			38 (67)			.27		
		Self-employed		5 (5)			1 (2)			4 (7)			.15		
		Retiree or unemployed		2 (2) 0 (0)			1 (2) 0 (0)			1 (1) 0 (0)			.84		
	**Marital status^f^, n (%)**														
		Single		24 (24)			9 (21)			15 (26)			.52		
		Married		63 (62)			27 (61)			36 (63)			.37		
		Divorced		12 (12)			7 (16)			5 (9)			.08		
		Widowed		2 (2)			1 (2)			1 (2)			.85		
**Clinical data**															
	**Diseases of the circulatory system**														
		**Coronary artery disease, n (%)**													
			1-vessel disease	31 (30)			13 (30)			18 (30)			.84		
			2-vessel disease	39 (37)			18 (41)			21 (34)			.81		
			3-vessel disease	35 (33)			13 (30)			22 (36)			.62		
		ST-segment elevation myocardial infarction or non–ST-segment elevation myocardial infarction, n (%)		59 (56)			26 (59)			33 (54)			.63		
		Cardiac arrhythmia, n (%)		6 (6)			4 (9)			2 (3)			.29		
		Arterial hypertension, n (%)		78 (74)			32 (72)			46 (75)			.55		
		Pulmonary embolism, n (%)		6 (5)			3 (7)			3 (5)			.53		
	**Endocrine, nutritional, or metabolic diseases, n (%)**														
		Obesity		28 (24)			13 (25)			15 (24)			.94		
		Type 2 diabetes mellitus		16 (15)			4 (9)			12 (19)			.18		
		Other		75 (71)			33 (75)			42 (68)			.59		
	(Hypo- and hyperthyroidism, n (%)				10 (9)			6 (11)			4 (6)			.36	
	Diseases of the musculoskeletal system and connective tissue, n (%)				20 (19)			9 (20)			11 (18)			.98	
	Depressive and adjustment disorders, n (%)				18 (17)			8 (18)			10 (16)			.99	
**Medication, n (%)**															
	Angiotensin converting enzyme inhibitor				54 (47)			22 (42)			32 (51)			.32	
	Statin				102 (97)			42 (95)			60 (98)			.15	
	β-Blocker				94 (89)			36 (82)			56 (91)			.36	
	Angiotensin-II receptor blocker				37 (32)			18 (34)			19 (30)			.67	
	Calcium channel blocker				22 (19)			8 (15)			14 (22)			.33	
	Anticoagulant				103 (98)			42 (95)			61 (100)			.45	
	Antiarrhythmic				1 (1)			1 (2)			0 (0)			.27	
	Diuretic				41 (39)			22 (50)			19 (31)			*.03*	
	Analgesic				13 (11)			6 (11)			7 (11)			.97	
	Antidepressant				5 (5)			2 (5)			3 (5)			.53	
	Diabetes medication				18 (17)			5 (11)			13 (21)			.12	

^a^Between-group comparison was performed using unpaired 2-sided *t* test or chi-square test.

^b^Not applicable.

^c^Italicization denotes statistical significance.

^d^A total of 19 participants did not provide their educational level (RehaPlus+ =12; usual care=7).

^e^A total of 5 participants did not provide their occupation level (RehaPlus+ =1; usual care=4).

^f^A total of 4 participants did not provide their marital status (RehaPlus+ =0; usual care=4).

### Primary Outcome

#### Physical Activity

**Figure 5:**
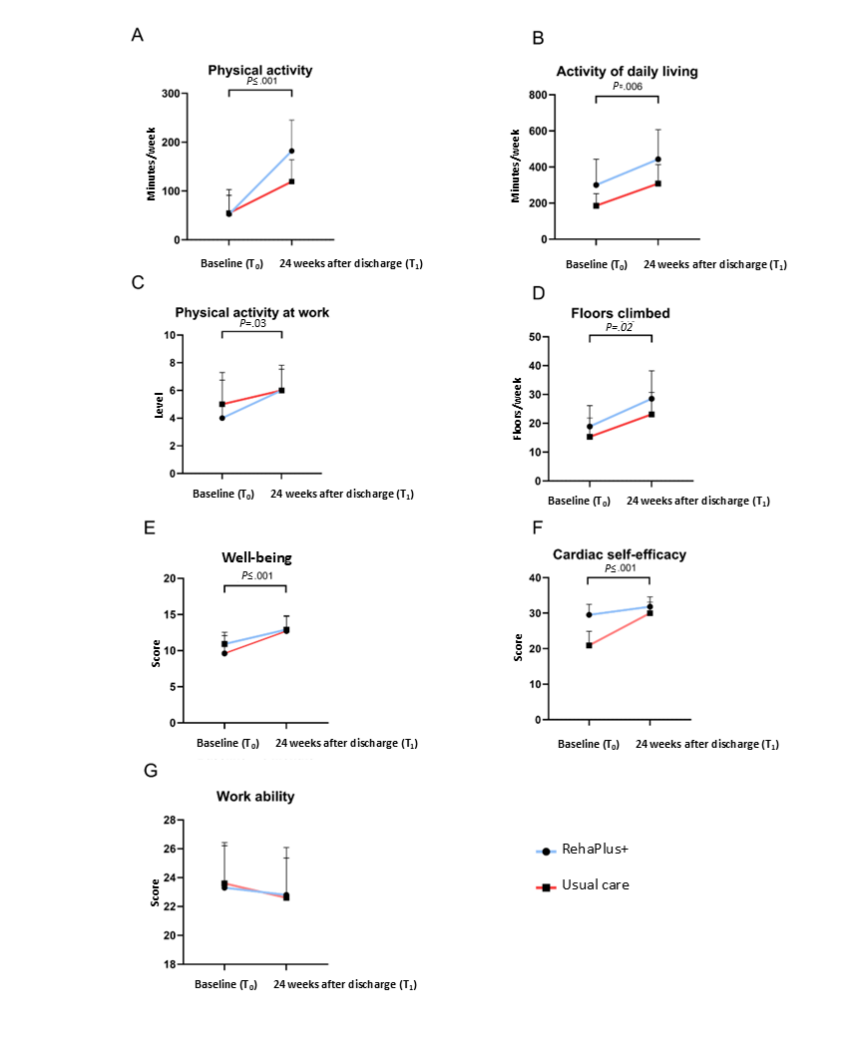
Changes in primary and secondary outcome measures over 6 months. All variables were assessed by questionnaire at T0 (start of phase II cardiac rehabilitation) and after 24 weeks. Overall, a significant increase at 6-month follow-up compared to baseline was detected in physical activity (*P*=.001), activities of daily living (*P*=.006), physical activity during work (*P*=.03), floors climbed weekly (*P*=.02), well-being (*P*=.001), and cardiac self-efficacy (*P*=.001) with no significant difference between groups. Data are presented as mean (SD). Differences between groups over time (RehaPlus+ vs usual care) were analyzed using 2-way ANOVA.

At 6 months after discharge from phase II CR, the RehaPlus+ group exhibited 182 (SD 208) minutes of PA per week, while the usual care group exhibited 119 (SD 175) minutes of PA per week (*P*=.12). To ensure statistical robustness and impartiality, outliers were removed (n=2), without any effect on the observed results (*P*=.10). Over time (T_0_ to T_1_) both groups combined showed a significant increase in PA per week, with an average increase of 92 (SD 211) minutes per week from prerehabilitation to 6-month follow-up (Figure 5A; *P*=.001). There were no significant effects of gender over time in terms of change in PA levels (*P*=.10). Notably, a responder analysis for PA revealed that 70% (31/44) of the RehaPlus+ participants were responders compared to 48% (29/61) of the usual care participants.

#### Activities of Daily Living

The RehaPlus+ group showed an ADL level of 443 (SD 538) minutes per week compared to the usual care group with 308 (SD 412) minutes per week at 6-month follow-up without significance (Table 2; *P*=.84). Of note, ADL levels were already higher in the RehaPlus+ group at baseline assessment. Removal of outliers (n=7) had no effect on the observed results (*P*=.37). Over time (T_0_ to T_1_), both groups combined showed a significant increase in ADLs per week from prerehabilitation to 6-month follow-up compared to T_0_ (Figure 5B; mean 131, SD 472 minutes; *P*=.006) without significant gender effects (*P*=.38). A responder analysis for ADLs revealed that 45% (20/44) of the RehaPlus+ participants were responders compared to 33% (20/61) of the usual care participants.

**Table 2:** Physical activity at 6-month follow-up by group^a^.

		Overall (N=105)	*P* value for time	RehaPlus+ (n=44)	Usual care (n=61)	*P* value for group	95% CI interaction	
**Physical activity**			*.001* ^b^					–127.2 to 11.2
	T0, mean (SD)	53.8 (164.8)		52.4 (127.1)	54.7 (188.3)	.99		
	T1, mean (SD)	145.5 (191.0)		181.85 (207.84)	119.20 (174.89)	.15		
	Δ, mean (SD)	91.7 (210.8)		129.5 (220.6)	64.5 (200.9)	.12		
	95% CI	–132.5 to –50.9		–196.5 to –62.4	–115.9 to –13.0			
**Activities of daily living**			*.006*					–200.3 to 75.9
	T0, mean (SD)	233.7 (365.9)		300.5 (469.1)	185.43 (262.2)	.31		
	T1, mean (SD)	364.6 (471.0)		442.79 (537.93)	308.27 (411.52)	.20		
	Δ, mean (SD)	131.0 (472.0)		142.3 (574.6)	122.8 (386.5)	.84		
	95% CI	–210.2 to –30.6		–317.0 to 32.4	–197.8 to –11.0			
**Levels of physical activity at work**			*.03*					–1.8 to 0.1
	T0, mean (SEM)	5 (9)		4 (9)	5 (9)	.08		
	T1, mean (SEM)	6 (6)		6 (6)	6 (6)	.96		
	Δ, mean (SEM)	1 (2)		1 (11)	0 (12)	.07		
	95% CI	–1.0 to –0.1		–1.8 to –0.2	–0.7 to 0.4			
**Floors climbed per week**			*.02*					–16.1 to 12.7
	T0, mean (SD)	16.8 (24.5)		18.9 (23.7)	15.3 (25.2)	.76		
	T1, mean (SD)	25.4 (30.7)		28.5 (32.0)	23.1 (29.8)	.56		
	Δ, mean (SD)	8.6 (36.4)		9.6 (36.4)	7.9 (36.8)	.82		
	95% CI	–15.6 to –1.5		–20.6 to 1.5	–17.3 to 1.6			

^a^Variables were assessed using the Bewegungs- und Sportaktivität questionnaire after 24 weeks. Differences between groups over time (RehaPlus+ vs usual care) were analyzed using 2-way ANOVA. Levels of physical activity at work: range 0 to 9 (higher=greater activity). T0: end of rehabilitation, T1: after 6 months, ∆, change from T0 to T1.

^b^Italicization denotes statistical significance.

### Secondary Outcomes

#### PA During Work

At 6-month follow-up (T_1_), the RehaPlus+ group and usual care group both reported an average level of PA at work of 6 (SEM 6) (; Table 2; *P*=.96), with no significant time×group interaction effect (*P*=.07). Compared with prerehabilitation (T_0_)_,_ an overall change in the level of PA during work (+ 1; *P*=.02) was observed at 6 months (Figure 5C).

#### Floors Climbed Weekly

At T_1_, the RehaPlus+ group showed an average of 28.5 (SD 32.0) floors climbed per week, while the usual care group had an average of 23.1 (SD 29.8) floors climbed per week (Table 2; *P*=.82). Overall, (both groups combined), there was a significant change in floors climbed weekly at 6-month follow-up compared to T_0,_ with an average increase of 8.6 (SD 36.4) floors climbed per week (Figure 5D; *P*=.02).

#### Psychological Well-Being

The preintervention scores for the RehaPlus+ and the usual care group showed an increase in psychological well-being, with the RehaPlus+ group changing from 9.6 (SD 8.1) to 12.7 (SD 7.0) postintervention and the usual care group changing from 10.9 (SD 6.4) to 12.9 (SD 7.2), without a significant time by group interaction (Table 3; *P*=.53). Compared to T_0,_ an overall change in well-being (mean 2.5, SD 7.6; *P*=.001) was observed at 6 months (Figure 5E).

**Table 3:** Change in well-being, cardiac self-efficacy, and work ability.

			Overall (N=105)		*P* value time		RehaPlus+ (n=44)		Usual care (n=61)		*P* value group		95% CI interaction
**Well-being**					*.001* ^b^								–3.9 to 2.0
	T0, mean (SD)	10.3 (7.1)				9.6 (8.1)		10.9 (6.4)		.61			
	T1, mean (SD)	12.9 (7.1)				12.7 (7.0)		12.9 (7.2)		.97			
	Δ, mean (SD)	2.5 (7.6)				3.1 (8.7)		2.1 (6.7)		.53			
	95% CI	–4.0 to –1.0				–5.7 to –0.4		–3.8 to –0.4					
**Cardiac self-efficacy**					*.001*								–0.5 to 13.1
	T0, mean (SD)	24.5 (14.1)				29.5 (9.8)		20.9 (15.6)		*.001*			
	T1, mean (SD)	30.8 (10.9)				31.8 (9.2)		30.0 (12.0)		.71			
	Δ, mean (SD)	6.3 (16.3)				2.3 (9.6)		9.1 (19.3)		*.03*			
	95% CI	–9.4 to –3.1				–5.2 to 0.6		–14.1 to –4.2					
**Work ability**					.66								–5.2 to 3.3
	T0, mean (SD)	23.3 (10.3)				23.0 (10.6)		23.6 (10.2)		.97			
	T1, mean (SD)	22.8 (10.8)				23.0 (11.0)		22.6 (10.8)		.98			
	Δ, mean (SD)	–1.0 (9.5)				0.0 (7.7)		–1.0 (10.6)		.66			
	95% CI	–1.5 to 2.6				−2.7 to 2.7		–2.1 to 4.0					

^a^The 5-item World Health Organization Well-Being Index (WHO-5), cardiac self-efficacy (CSE), and Work Ability Index (WAI) were assessed by a questionnaire at baseline (start of phase II cardiac rehabilitation, T0) and after 24 weeks (T1). Δ indicates the mean individual difference between T0 and T1. Differences between groups over time (RehaPlus+ vs usual care) were analyzed using 2-way ANOVA. WHO-5: range 0 to 25 (higher=greater well-being), WAI: range 7 to 49 (higher=improved work ability), and CSE: range 0 to 100 (higher=greater well-being).

^b^Italicization denotes statistical significance.

#### CSE Overview

The baseline values of CSE were significantly different, with the RehaPlus+ group starting with a significantly higher value (*P*=.001). The preintervention scores for the RehaPlus+ and the usual care group showed an increase in CSE, with the RehaPlus+ group changing from 29.5 (SD 9.8) to 31.8 (SD 9.2) postintervention and the usual care group changing from 20.9 (SD 15.6) to 30.2 (SD 12.0), with a significant time by group interaction (Table 3; *P*=.03). At the 6-month follow-up, there was a significant improvement in CSE across all patients compared to baseline (T0), with an average increase of 6.3 points (SD 16.3; Figure 5F; *P*=.001).

#### Work Ability

At T_0_, the RehaPlus+ group had an average Work Ability Index score of 23.0 (SD 10.6) at baseline and an average score of 23.0 (SD 11.0) at T_1_, while the usual care group decreased from 23.6 (SD 10.2) to 22.6 (SD 10.8); however, there was no significant difference between the 2 groups over time (Table 3; *P*=.66).

#### Health-Related QoL

**Figure 6:**
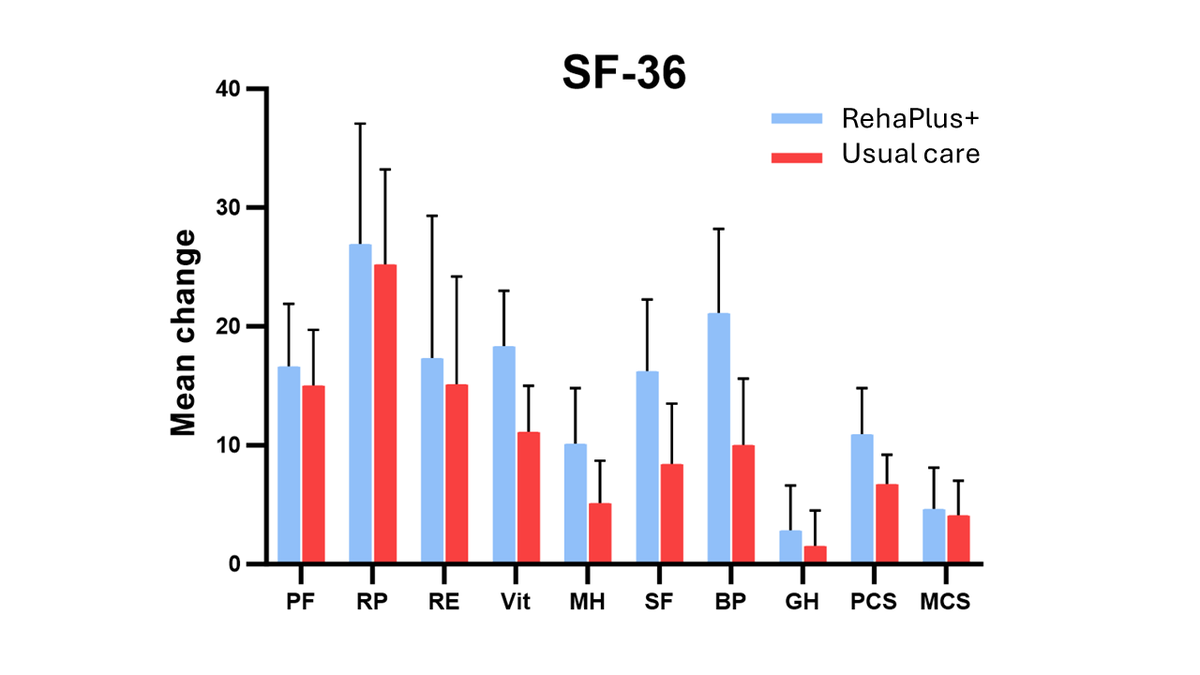
Health-related quality of life assessed by 36-item Short Form Survey (SF-36) questionnaire. At baseline, SF-36 scores were comparable between both groups, although the usual care group had a higher mental component summary (MCS) score (*P*=.03). Both groups increased their preintervention score, with no difference observed between the 2 groups (P≥.362). Data are presented as 95% CI; changes over time between groups (time×group interaction) were calculated using general linear model. SF-36 score range: 0-100 (higher=greater well-being). BP: bodily pain; GH: general health; MH: mental health; PCS: physical component summary; PF: physical functioning; RE: role emotional; RP: role physical; SF: social functioning; Vit: vitality.

The SF-36 scores were comparable between both groups at T_0;_ however, the usual care group started with a higher MCS score (*P*=.03). Over time (T_0_-T_1_), health-related QoL changed significantly during phase III CR over both groups combined (RehaPlus+: MCS+14% and PCS+18%; usual care: MCS+12% and PCS+17%; *P*=.05), with no difference between the groups (*P*=.36; Figure 6).

## Discussion

### Principal Findings

The aim of this study was to evaluate the efficacy of the multimodal, behavioral change–based maintenance program RehaPlus+ and to determine its noninferiority to the German usual care. In brief, the main findings of the study are that (1) the RehaPlus+ program, which uses BCTs and theories delivered partly via eHealth, was found to be noninferior to the usual care program; (2) both interventions induced a comparable and significant increase in PA during work, floors climbed weekly, psychological well-being, CSE, work ability, and health-related QoL; and (3) the implementation of the RehaPlus+ messaging system was perceived as user-friendly by the involved case managers. The analysis revealed that there were no significant differences between the RehaPlus+ and usual care group across outcome measures.

RehaPlus+ uses a range of established BCTs such as goal setting, action planning, and motivational and coping strategies to empower patients with cardiac conditions to make positive lifestyle changes. BCTs are designed to target and modify a specific behavior that plays a role in overall health and well-being. Goal setting as a central component likely empowered participants to establish clear and attainable objectives related to their desired outcomes, as research highlights the effectiveness of these techniques in promoting PA in patients with cardiac conditions [43]. Action planning is crucial in the initial phases of rehabilitation, while coping strategies are hypothesized to facilitate the long-term maintenance of behavior change [44]. Coping strategies may have helped RehaPlus+ participants to identify and address barriers, enabling them to develop strategies for overcoming challenges and sustain their progress. This is supported by the observation that individuals with higher levels of coping planning postdischarge are more likely to engage in increased levels of exercise [45]. Coping planning is a self-regulation strategy designed to anticipate and counteract personal risk situations that threaten intended behavior. It creates a mental connection between potential risks and appropriate coping responses [45]. It has been demonstrated that people who effectively plan for high-risk situations, such as exercise relapse, are less likely to give in to these challenges [46]. This approach is aligned with techniques used in cognitive behavioral therapy, where anticipating and preparing for potential challenges helps manage unwanted behaviors. While action planning facilitates task execution, coping planning focuses on avoiding distractions. The effectiveness of coping planning increases over time with experience, as it relies on an individuals’ understanding of their personal risk situations and responses. This strategy is crucial for maintaining focus on long-term goals and preventing unwanted behaviors [45]. Coping planning interventions are found to be effective, especially when participants receive support in developing their coping plans [47]. This approach is similar to the one used in the RehaPlus+ program, where guided assistance in coping plan formulation is a key component.

In RehaPlus+, the implementation of BCTs may have contributed to the development of healthy exercise habits and enhanced self-efficacy for lifestyle changes. However, it is crucial to assess the respective contributions of the technology-driven message delivery system and the support provided by the case managers to the overall success of the intervention. The case manager met the patient in a face-to-face session and helped to create an action plan and set goals, considering potential barriers. The case manager then developed a tailored message chain for the patient, considering the discussed goals and planned actions. In addition, the case manager contacted the individuals with cardiac conditions during 2 phone calls to adjust the timing or content of the messages based on any changes in their activities or work schedules. This aspect represents a common challenge in studies of this nature, and the individual impact of each component on the outcomes cannot be differentiated.

According to the Health Action Process Approach [31], the maintenance of health behavior requires specific input, including: (1) action planning, to specify situation parameters (“when” and “where”) and a sequence of action (“how”) to implement intended behavior; (2) action control, to help sustaining the behavioral change; and (3) coping planning as a self-regulatory strategy or alternative behavior to overcome barriers. Therefore, RehaPlus+ case managers suggested practical solutions to everyday barriers and motivated participants to incorporate “what-if” sentences to their individual action plans. These sentences focus on potential obstacles and simultaneously identified solutions, such as “If it’s raining during my scheduled outdoor workout, I will move indoors and complete my exercise routine.” RehaPlus+ aimed to motivate patients with CAD toward increased, self-organized PA as well as increased ADLs through targeted lifestyle adjustments and health-promoting behavior in everyday life. In contrast, the usual care program focuses on promoting PA through regular appointments for guided group-based exercise sessions. However, despite its targeted focus on PA, usual care did not demonstrate superiority over RehaPlus+. Both programs were effective in promoting PA, suggesting that different methodologies in rehabilitation can be equally successful in achieving similar outcomes in PA enhancement.

With regard to ADLs, a recent meta-analysis reported significant effects of different eHealth interventions in increasing walking steps in older adults [48], suggesting that eHealth has the potential to effectively enhance everyday activities with minimal barriers and versatility in practice. Our data support these findings in that the RehaPlus+ group showed an increase in ADLs, likely caused by the fact that RehaPlus+ was designed to stimulate a broader range of daily activities. This observation is consistent with previous research [49] emphasizing the significant impact of BCTs on habit formation, which play a crucial role in maintaining regular exercise routines for cardiovascular health promotion. The effects of RehaPlus+ may also relate to the personalized approach, which considered individual barriers. Tailoring the intervention to individual characteristics and preferences is a crucial aspect of ensuring the success of the rehabilitation program. By focusing also on implementation, RehaPlus+ sought to maximize the effectiveness of the BCTs, aligning them with the unique needs of each patient. This approach is in line with the principle that the success of behavior change interventions is significantly influenced by how well they are adapted to individual patient profiles [50], ensuring that the strategies used are not only theoretically sound but also practically relevant and effective in the specific context of each patients’ rehabilitation journey. However, it is possible that the combination of human intervention and personal contact during the maintenance period together with the use of technology may have contributed to the observed treatment effect synergistically. RehaPlus+ integrates a personalized eHealth intervention with behavioral change concepts, emphasizing healthy lifestyle changes also supported by case managers. It is likely that the personal contact enhances the value and trustworthiness of the messages, further enhancing the effectiveness of the intervention. In addition, technology enhances accessibility, convenience, and scalability, allowing for continuous engagement at home beyond traditional center visits. Further studies are warranted to delineate the relative contributions of human interaction and technology in driving treatment outcomes. For instance, future investigations could explore the impact of reducing the duration of personal contact or modifying the intensity of the message component.

The effectiveness of BCTs is assumed to depend on their appropriate implementation, which should take into account individual characteristics and preferences. The improvements in health-related QoL, CSE, and overall well-being may have likely played a role in adherence to the respective programs.

With respect to the participants’ baseline characteristics, it seems important to note that 94% (99/105) of the participants did not have qualifications extending beyond high school level. Of note, the RehaPlus+ group had a higher percentage of participants (15/44, 33%) with an educational level exceeding high school. While it is unlikely that these differences may have influenced the outcomes, the finding underscores that eHealth interventions should be adaptable to the individual participant and potential hurdles for patients with lower literacy should be reduced. In general, it is crucial that eHealth solutions, such as mobile apps, should be easily understandable and accessible to all patients, regardless of their educational background. In the context of gender-sensitive care, caregiving can exert distinct mental and physical health effects depending on gender roles and societal expectations. A gender-sensitive approach allows for tailored interventions and support systems, acknowledging and mitigating these unique health impacts experienced by caregivers of different genders.

The use of eHealth programs such as RehaPlus+ provides several advantages, including cost-effectiveness [51]. In this case, the outpatient usual care program IRENA incurs costs of approximately €770 (US $850) per patient for 24 weeks. In contrast, the eHealth program RehaPlus+ costs approximately €540 (US $600) per patient for the same duration [52]. However, RehaPlus+ is scalable, and the costs in other countries will depend on local conditions and circumstances. In addition, similar outcomes can be achieved with lower costs, reduced resource use, and less staff burden.

Recent systematic reviews have highlighted the cost-effectiveness of cardiac telerehabilitation in a broader context, showing that eHealth interventions hold the capacity to reach a broader and more diverse audience at a lower cost [53,54]. Such programs can overcome geographical barriers, enhancing accessibility for individuals situated in remote or underserved areas. Furthermore, eHealth programs can be structured for self-administration, reducing the demand for extensive human resources and subsequently lowering operational expenses. However, for a comprehensive assessment, a detailed cost-effectiveness analysis of RehaPlus+ would be required to compare the expenses associated with implementing eHealth programs against the benefits in terms of health outcomes and reduced health care use.

### Limitations

The self-reported nature of PA is one of the major limitations of this study. This method relies on subjective assessments and memory recall, which could lead to biases in reporting participants’ actual activity levels, even if likely comparable between both groups. The study experienced considerable loss to follow-up rates, primarily due to difficulties in obtaining questionnaire-based outcome data 6 months after the intervention. Although the observed rates were comparable between both groups and equal to previous eHealth and non–technology-based interventions for behavior maintenance [55-57], it is crucial to thoroughly examine that motivation is a relevant aspect in eHealth approaches, and there is a risk that eHealth interventions may ultimately leave only highly compliant patients. Patients who drop out of eHealth interventions may require different incentives and motivations to stay engaged and achieve their health goals in the long term. To effectively support individuals in earlier stages of change, it may be essential to first implement other interventions aimed at promoting intrinsic motivation for change. These interventions may include motivational interviewing, psychoeducation about the risks and benefits of behavior change, and building self-efficacy through goal setting and reinforcement. By addressing these factors, individuals in stage 1 or 2 (according to the TTM) can be supported in developing the internal drive necessary for sustained behavior change [28]. In addition, it is important to acknowledge that baseline data from 11 participants were unavailable for analysis. These participants were prematurely discharged from their rehabilitation due to COVID-19 infections, hindering the completion of baseline assessments. Furthermore, outcomes were measured by questionnaires, and no objective measurements were used.

### Conclusions

This study demonstrates that the behavioral change–based eHealth maintenance program RehaPlus+ was equally effective as the standard German usual care in promoting regular ADLs and PA in patients with CAD. This achievement can be attributed, in part, to the successful use of BCTs within the RehaPlus+ program. This study underscores the importance of evidence-based BCTs in eHealth interventions and highlights the need for personalized eHealth intervention strategies. Future research should refine BCT implementation in eHealth programs to improve health behaviors across diverse populations.

## Appendix

Multimedia Appendix 1 CONSORT-EHEALTH (Consolidated Standards of Reporting Trials of Electronic and Mobile Health Applications and Online Telehealth) version 1.6.

## Abbreviations

ADL: activity of daily living
BCT: behavior change technique
CAD: coronary artery disease
CONSORT: Consolidated Standards of Reporting Trials
CR: cardiac rehabilitation
CSE: cardiac self-efficacy
IRENA: Individualized Rehabilitation Aftercare in Post-Acute Treatment
MCS: mental component summary
PA: physical activity
PCS: physical component summary
QoL: quality of life
TE: typical error
TTM: transtheoretical model

## Data Availability

The data sets generated and analyzed during this study are available from the corresponding author on reasonable request.
